# Excessive Reactive Iron Impairs Hematopoiesis by Affecting Both Immature Hematopoietic Cells and Stromal Cells

**DOI:** 10.3390/cells8030226

**Published:** 2019-03-08

**Authors:** Hirokazu Tanaka, J. Luis Espinoza, Ryosuke Fujiwara, Shinya Rai, Yasuyoshi Morita, Takashi Ashida, Yuzuru Kanakura, Itaru Matsumura

**Affiliations:** 1 Department of Hematology and Rheumatology, Kindai University Faculty of Medicine, Osaka-sayama 589-8511, Japan; luis@med.kindai.ac.jp (J.L.E.); r-fujiwara@med.kindai.ac.jp (R.F.); rai@med.kindai.ac.jp (S.R.); moriyasu@med.kindai.ac.jp (Y.M.); ashida@med.kindai.ac.jp (T.A.); i.matsu@med.kindai.ac.jp (I.M.); 2 Department of Hematology and Oncology, Osaka University Graduate School of Medicine, Suita, Osaka 565-0871, Japan; kanakura@bldon.med.osaka-u.ac.jp

**Keywords:** hematopoiesis, iron overload, hematopoietic stem cells, stromal cells, oxidative stress

## Abstract

Iron overload is the accumulation of excess iron in the body that may occur as a result of various genetic disorders or as a consequence of repeated blood transfusions. The surplus iron is then stored in the liver, pancreas, heart and other organs, which may lead to chronic liver disease or cirrhosis, diabetes and heart disease, respectively. In addition, excessive iron may impair hematopoiesis, although the mechanisms of this deleterious effect is not entirely known. In this study, we found that ferrous ammonium sulfate (FeAS), induced growth arrest and apoptosis in immature hematopoietic cells, which was mediated via reactive oxygen species (ROS) activation of p38MAPK and JNK pathways. In in vitro hematopoiesis derived from embryonic stem cells (ES cells), FeAS enhanced the development of dysplastic erythroblasts but inhibited their terminal differentiation; in contrast, it had little effect on the development of granulocytes, megakaryocytes, and B lymphocytes. In addition to its directs effects on hematopoietic cells, iron overload altered the expression of several adhesion molecules on stromal cells and impaired the cytokine production profile of these cells. Therefore, excessive iron would affect whole hematopoiesis by inflicting vicious effects on both immature hematopoietic cells and stromal cells.

## 1. Introduction

Iron is an essential component of numerous proteins and enzymes that are involved in oxygen transport and storage (hemoglobin, myoglobin), electron transfer and drug metabolism (cytochrome c), and signal transduction (nitric oxide synthases) [1,2]. Excessive concentrations of iron, however, are toxic to cells, thus almost all organisms have developed tightly regulated mechanisms to maintain iron homeostasis [1,3]. As mammals lack an active system for iron excretion, iron homeostasis is maintained by regulating intestinal iron absorption [4]. In healthy subjects, iron is absorbed from the diet through the epithelium of the upper small intestine at a rate of 1 to 2 mg/day, and is lost at an almost equivalent rate through sloughed epithelial cells and blood cells [1,4]. The liver hormone hepcidin binds to ferroportin transmembrane protein during states of excess iron or inflammation thus reducing the amount of iron released into the circulation by enterocytes and macrophages thus controlling iron absorption, recycling, and storage [5].

Iron overload (hemochromatosis) can be primary or secondary. Primary hemochromatosis is a genetic disorder associated with mutations of the *HFE* gene and other genes that alter proteins involved in the regulation of intestinal iron absorption. On the other hand, secondary iron overload is caused by any other disorder associated with iron accumulation in the organs, is most commonly induced after repeated red blood cell transfusions such as in patients with thalassemia, sickle cell disease, myelodysplastic syndromes, and other acquired and inherited refractory anemias [4,6].

In both cases, when the plasma transferrin pool is highly saturated by excessive iron, non-transferrin bound iron (NTBI) accumulates in the plasma, and a portion of this plasma NTBI, which is called labile plasma iron (LPI), is highly toxic to cell membranes [7,8]. Cellular uptake of NTBI occurs independently of transferrin receptor 1 (TFR1), likely via 2^+^ metal channels such as DMT1, and NTBI accumulates in the cells as free iron in labile iron pools (LIPs) [6].

Iron cycles between ferric (Fe^3+^) and ferrous (Fe^2+^) forms through the donation or acceptance of an electron [3]. These reactions yield reactive oxygen species (ROS) such as hydroxyl radicals (OH-), superoxide (O2^−^), and hydrogen peroxide (H_2_O_2_); among these, hydroxyl radicals are highly toxic for cells and cause oxidation of lipids, proteins, and DNA, thereby inducing cell death and tissue damage [9]. Excessive iron induces cell death in various cell lines and under various culture conditions via multiple cell death mechanisms including apoptosis, necroptosis and ferroptosis, all of which are, at least in part, dependent on iron or iron-dependent ROS [10].

In the early stage of iron overload, iron accumulates in specific tissues, which is dependent on the disease and/or cause. For example, in hereditary hemochromatosis, iron deposition is initially observed in hepatocytes [11], while excessive iron from blood transfusions accumulates predominantly in the reticulo-endothelial system [1]. However, in the late stage of iron overload, excessive iron accumulates in and injures multiple types of cells and tissues, and its clinical toxic effects are mainly observed in the heart, liver, and endocrine system [6,12]. Notably, mouse models have shown that erythropoiesis is not severely impaired in hemochromatosis and indeed have documented higher hemoglobin values associated with iron overload [13] and patients with hereditary hemochromatosis tend to have increased erythrocytes and hemoglobin content [14].

A substantial fraction of patients with hematologic diseases such as aplastic anemia, myelodysplastic syndromes (MDS), and thalassemia exhibit iron overload, though the mechanism underlying iron overload varies depending on the disease. For example, aplastic anemia patients show iron overload due to a defect in iron utilization, while in MDS and thalassemia patients, iron accumulation is a result of increased iron absorption [15,16].

Excessive iron accumulates in the bone marrow including the hematopoietic cells compartment where it induces the generation of ROS, thereby injuring hematopoietic cells [9,17]. Consistent with these observations, iron chelation therapy is associated with dramatic improvements in erythropoiesis, granulopoiesis and megakaryopoiesis in a significant proportion of patients with hematopoietic diseases [18,19,20]. In addition, transferrin may also function to prevent or reduce iron accumulation in tissues, and this agent, in the form of apotransferrin, is under investigation for its therapeutic potential to prevent disease progression in thalassemia [21].

In the hematopoietic system, iron homeostasis regulated by the FBXL5–IRP2 axis is integral to the maintenance of HSCs, and ablation of FBXL5 specifically in the hematopoietic system of mice results in cellular iron overload in HSCs along with impaired repopulation capacity. FBXL5-deficient HSCs manifested oxidative stress, and increased exit from quiescence and eventual exhaustion [22,23]. In addition, increased OS has been documented in bone marrow (BM) cells of patients with iron overload coupled with impaired hematopoietic function, which was partially ameliorated in the presence of antioxidants or iron chelators [24]. It must be noted however that the molecular mechanisms underlying hematopoietic suppression by systemic iron overload have not been fully elucidated and the potential effects of cellular iron overload on bone marrow niche, stromal cells and on their interaction with HSC remain unknown.

In this study, we examined the effects of iron overload on the function of primary hematopoietic cells and stromal cell lines, and found that iron overload impairs normal hematopoietic cells and modifies the function of stromal cells, indicating that iron overload impairs the whole hematopoietic system.

## 2. Materials and Methods

### 2.1. Reagents and Antibodies (Abs)

Recombinant murine stem cell factor (mSCF), murine interleukin-6 (mIL-6), murine IL-3 (mIL-3), murine G-CSF (mG-CSF), human thrombopoietin (hTPO), and human erythropoietin (hEPO) were provided by Kirin Brewery Company (Tokyo, Japan). Murine FLT3 ligand (mFL) was purchased from PEPROTECH (London, UK). Ferrous ammonium sulfate (FeAS) and L-ascorbic acid were purchased from Sigma-Aldrich, Inc (St. Louis, MO, USA). Biotinylated anti-lineage (Lin) Abs against Gr-1 (RB6-8C5), B220 (RA3-6B2), CD3e (145-2C11), Mac1 (M1/70), Ter119 (TER119), fluorescein isothiocyanate (FITC)-labeled anti-Sca-1 (D7), allophycocyanine (APC)-labeled anti-c-Kit (2B8), phycoerythrin (PE)-labeled CD71, PE-labeled phospho-p38MAPK (pT180/Y182) (36), and Abs against ICAM1, E-cadherin (36), VE-cadherin (11D4.1), E-selectin (10E9.6), and PECAM1 (MEC13.3) were purchased from BD Biosciences (San Jose, CA, USA). Phospho-SAPK/JNK (pT183/pY185) (G5) was purchased from Cell Signaling Technology Inc. (Danvers, MA, USA). Calcein-acetoxymethyl ester (Calcein–AM), Redoxsensor^TM^ RedCC-1, Hoechst33342, Alexa Fluor 488 anti-mouse IgG, and Alexa Fluor 546 anti-rabbit IgG were purchased from Invitrogen (Carlsbad, CA, USA). Annexin-V plus propidium iodide (Annexin V/PI) assay (BD-PharMingen, San Diego, CA, USA) was used to determine the type and stage of cell death with flow cytometry.

### 2.2. Cell Lines and Culture

The MS5 murine bone marrow (BM) stromal cell line was acquired from Dr. Takafumi Yokota, Department of hematology and Oncology of Osaka University and was maintained in DMEM supplemented with 10% fetal bovine serum (FBS). The murine embryonic cell lines OP9 cells and E14tg2a cells were provided by Dr. Takumi Era of the Institute for Frontier Medical Sciences, Kyoto University. OP9 cells were cultured in αMEM medium supplemented with 20% FBS and E14tg2a cells were cultured in DMEM + 2 mM Glutamine + 1% NEAA + 0.1 mM 2-Mercaptoethanol + 1000 U/mL mouse inhibitory factor (LIF) + 1.5% sodium bicarbonate + 10% FBS.

### 2.3. Purification of Murine Lineage^−^Sca1^+^c-Kit^+^(LSK) Cells

Murine BM cells were harvested from 8- to 10-week-old C57BL/6 mice, and mononuclear cells (MNCs) were isolated by density gradient centrifugation. After staining with biotinylated anti-Lin Abs, FITC-conjugated anti-Sca-1 Ab, APC-conjugated anti-c-Kit Ab and streptavidin-PE-cy7, LSK cells were sorted by FACS Aria (BD Biosciences).

### 2.4. ATP Assay

LSK cells were plated in 96-well plate at a density of 1 × 10^3^ cells/well and maintained at 37 °C in a 5% CO_2_ incubator. After 4 and 7 days of initial plating, intracellular ATP levels in cultured cells were measured by CellTiter-Glo Luminescent Cell Viability Assay (Promega Co., Madison, WI, USA) according to manufacturer’s instructions.

### 2.5. Flow Cytometry

Cell surface markers were analyzed by FACS Canto II (BD Biosciences). The DNA content of cultured cells was examined by propidium iodide (PI) staining. FACS data were analyzed by FlowJo software (TreeStar, Ashland, OR, USA). The phosphorylation states of p38MAPK and JNK were analyzed by Phosflow^TM^ Technology (BD Biosciences). Briefly, cells were fixed with Fixation buffer, permeabilized with Perm buffer II, and stained with phosphospecific Abs. Flow cytometry was performed on FACS Canto II equipped with 488 and 633 nm lasers.

### 2.6. Coculture with MS5 Cells

LSK cells (100 cells/well) or BM MNCs (1 × 10^4^ cells/well) were seeded onto a pre-established monolayer of MS5 cells in 24-well tissue culture plates and cultured in αMEM supplemented with 10% FCS with indicated cytokines. After 7 days of culture, non-adherent and adherent hematopoietic cells were collected by gentle pipetting and EDTA-trypsin treatment, respectively. Contaminating MS5 cells were removed by a 30-min incubation in a tissue culture flask as adherent cells. Collected hematopoietic cells were counted and subjected to flow cytometric analysis and colony assays.

### 2.7. Cobblestone-Like Area Forming Cell (CAFC) Assay

BM MNCs (1 × 10^4^/well) were plated on mitotically inactivated MS5 cells (inactivated by pretreatment with mitomycin C) in six-well plates. After 3 or 4 days, the number of cobblestone-like areas was counted by microscopy. To assess the effects of iron overload on stromal cells, in some experiments, mitotically inactivated MS5 cells were cultured in six-well plates in the presence or absence of 50 μM of FeAS for 48 hours and after removing the culture medium, LSKs (1 × 10^4^/well) were added and co-cultured for another 48 hours with or without FeAS (50 μM) and the number of cobblestone-like areas was counted by microscopy.

### 2.8. OP9 System to Develop Hematopoietic Cells from ES Cells

To induce differentiation into hematopoietic cells, ES cells were deprived of leukemia inhibitory factor (LIF) and seeded onto confluent OP9 cells in six-well plates at a density of 1 × 10^4^ cells/well in αMEM supplemented with 10% FBS. After 4.5 days, cultured cells were harvested by Cell dissociation buffer (Gibco, Rockville, MD, USA), and Flk-1^+^ cells were sorted and replated onto OP9 cells at a density of 1 × 10^4^ cells/well in a six-well plate or 6 × 10^4^ cells in a 10-cm dish, and cultured under the indicated conditions.

### 2.9. Colony Assays

Cultured MNCs (1 × 10^4^ cells/35-mm dish) were seeded in methylcellulose media containing hEPO (3 U/mL), mIL-3 (10 ng/mL), hIL-6 (10 ng/mL), and mSCF (50 ng/mL) (M3434, Stem Cell Technologies, Vancouver, BC, Canada) and the colonies formed were evaluated after 10 to 14 days of culture.

### 2.10. Measurement and Detection of Intracellular LIP

Cellular LIP was measured with the fluorescent metalosensor Calcein-AM as previously reported [25]. In brief, cells were incubated with 250 nM Calcein-AM for 5 min at 37 °C in α-MEM, washed twice with saline, and resuspended at a density of 1 × 10^6^/mL in α-MEM containing 1% bovine serum albumin at room temperature. Cells were then washed and resuspended in Na-HEPES (20 mM) buffered saline (HBS; 145 mM NaCl, pH 7.2, 37 °C). Fluorescence intensity (488 excitation, 517 nm emission) was measured with a continuous mode using FACS Canto II (BD Biosciences).

### 2.11. Fluorescence Microscopy

Cells stained with Calcein-AM, RedCC-1, and Hoechst 33,342 were analyzed with a Zeiss Laser Scanning microscopy system (Zeiss LSM5 PASCAL, Obercochen, Germany).

### 2.12. Cytokine Assays for Stromal Conditioned Medium

Cytokine levels in the culture medium were measured by the RayBio Mouse Cytokine Antibody Array C series (RayBiotech, Inc. Norcross, GA, USA) according to the manufacturer’s instructions. In brief, the cytokine array membrane was incubated with 1.2 mL culture medium obtained after 48 h culture with or without FeAS, and subsequently washed with buffer I and buffer II. The membrane was then incubated with cytokine antibodies for 1 h, followed by HRP-labeled streptavidin incubation for 1 h. Reactive spots were visualized by enhanced chemiluminescence (ECL) (Amersham Pharmacia Biotech UK Limited, Piscataway, NJ, USA) with exposure to X-ray film and the intensity of spots (corresponding to individual cytokines and factors) were determined by densitometry as previously reported [26].

### 2.13. Statistical Analysis

Data were presented as mean ± SEM or mean ± SD. The statistical significance of the data was determined by the Mann–Whitney *U* test or Student’s *t* test. Data were analyzed using the Graph Pad prism 6 software package (San Diego, CA, USA). When *p* values were less than 0.05 they were considered statistically significant.

## 3. Results

### 3.1. FeAS Induces Growth Arrest and Apoptosis in Murine LSK Cells

**Figure 1:**
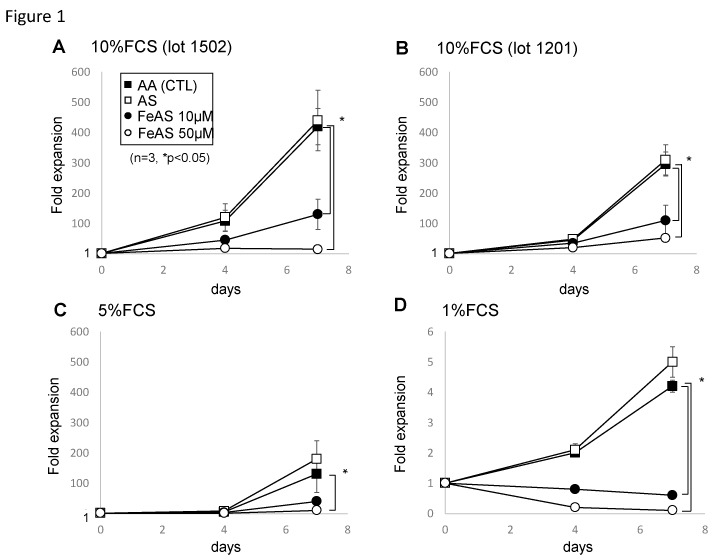
Ferrous ammonium sulfate (FeAS) interfered with the growth of LSKs in the liquid culture system. Lineage^−^Sca1^+^c-Kit^+^(LSK) cells isolated from murine bone marrow (BM) were cultured in αMEM medium containing hematopoietic cytokines (100 ng/mL each) and supplemented with 10% FBS (**A**,**B**), 5% fetal bovine serum (FBS) (**C**), or 1% FBS (**D**) in the absence (untreated (AA CTL) or the presence of 10 μM (FeAS 10 μM) or 50 μM (FeAS 50 μM) ferrous ammonium sulfate, or with ammonium sulfate (AS). Total viable cell numbers were determined using an ATP assay. Data shown indicate the mean ± SD of summarized data obtained from three independent experiments (* *p* < 0.05).

To examine the effects of iron overload on hematopoiesis, we first cultured murine LSK cells with several cytokines in the presence of 2 mM ascorbic acid (to maintain iron in its ferrous state) and two different concentrations of FeAS [10 μM (39.2 μg/dL) or 50 μM (196 μg/dL)]. These concentrations of FeAS are slightly higher than the level observed in human sera (normal ranges 65 to 176 µg/dL in men and 50 to 170 µg/dL in females with a 35% of transferrin saturation) [27,28]. As shown in Figure 1A,B, the growth of LSK cells was significantly inhibited by 10 μM of FeAS and was completely blocked by 50 μM of FeAS, however, these effects varied depending on the lot of FBS utilized to support the culture of LSK cells, with more severe inhibitory effects observed when the culture medium was supplemented with FCS lot 1502 (Figure 1A).

We next cultured LSKs in medium supplemented with low percentages of FBS (5% or 1%) to exclude the possibility that the presence of a carrier protein, such as transferrin present in FBS, could influence the results of the experiments. As shown in Figure 1C,D, the growth inhibition of the LSKs caused by FeAS-treatment was even more evident when cells were cultured in the medium supplemented with lower concentrations of FBS. Moreover, ammonium sulfate (AS), the salt included in the iron preparation used in these studies, did not show growth inhibitory effects in any these experiments, indicating that non-protein-bound, free iron interfered with the growth of LSKs in the liquid culture system.

**Figure 2:**
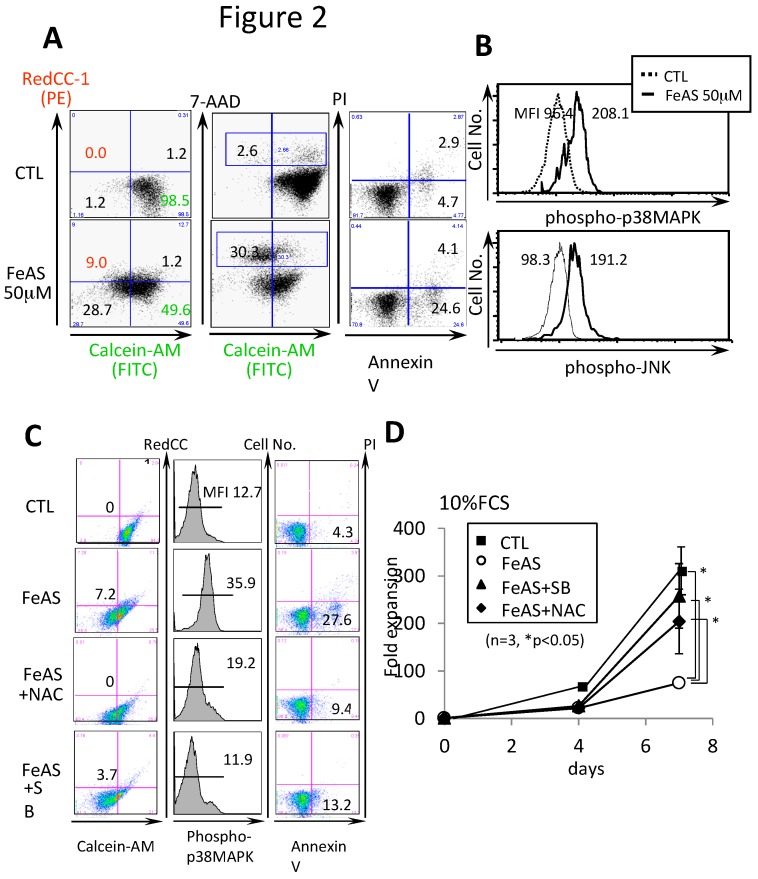
FeAS induces growth inhibition and cell death through reactive oxygen species (ROS)-activated p38MAPK. (**A**) After 72 h of culture with or without FeAS, LSK cells were stained with Calcein-AM and Red CC-1 and subjected to flow cytometry analyses. Intracellular labile iron and total ROS were detected with Calcein-AM and Red CC-1, respectively. FeAS-induced cell death was assessed as Calcein-AM low/negative and 7-AAD positive staining. The percentage of 7-AAD positive dead cells is indicated by the number in the quadrant. (**B**) After 72 h of culture with or without FeAS, cells were stained with specific antibodies, and threonine and tyrosine phosphorylation of p38MAPK and JNK/SAPK was examined by FACS. (**C**) Effects of the anti-oxidant (N-Acetyl-l-cysteine, NAC) and p38MAPK inhibitor (SB203580, SB) on ROS-accumulation (left panel), phosphorylation of p38MAPK (middle panel), and the type and stage of cell death (right panel) was examined by FACS. LSK cells were cultured with 50 μM of FeAS in combination with either 100 μM NAC or 10 μM SB203580 for 72 h. The percentage of Calcein-AM low/RedCC1+ fraction, mean fluorescence intensity (MFI) of phosphor-p38MAPK, and annexinV+/PI- apoptotic fraction are indicated, respectively. (**D**) Effects of NAC and SB on FeAS-induced growth inhibition of LSKs were evaluated. Data is shown as the means ± SD of summarized data obtained from three independent experiments (* *p* < 0.05).

Next, we utilized flow cytometry analysis to assess the amount of iron and reactive oxygen species (ROS) in cultured LSKs using the fluorescence dyes, Calcein-AM and RedCC-1 respectively. Calcein-AM is a green fluorescent iron-chelating agent, and its fluorescent intensity is reduced by iron binding. RedoxSensor™ Red CC-1 stain is a probe whose fluorescence localization appears to be based on a cell’s cytosolic redox potential. Red CC-1 passively enters live cells, and is oxidized in the cytosol to a red-fluorescent product, which then accumulates in the mitochondria. As shown in Figure 2A, although neither iron nor ROS was detectable in 98.5% of the cells in the control culture (CTL), iron accumulated in 37.7% (28.7% + 9.0%) of FeAS-treated cells. Additionally, ROS were detected in a significant proportion of FeAS-treated cells.

We next examined the states of ROS-responsive molecules in FeAS-treated LSK cells. Although we did not detect ERK1/2 or RelA (a subunit of NF-κB, p65) activation in LSK cells in response to FeAS treatment (data not shown), p38MAPK and JNK were both activated in cells exposed to FeAS (Figure 2B). Notably, the activation p38MAPK and JNK is known to induce cell death in hematopoietic stem cells, especially in the context of cellular stress [29,30]. In agreement with this result, 30.3% of FeAS-treated cells were positive for the dead cell marker 7-AAD compared to 2.66% in the CTL culture (Figure 2A).

We next analyzed the roles of ROS and p38MAPK in FeAS-induced cell death. When ROS were eliminated by treatment with an anti-oxidant, N-acetyl-l-cysteine (NAC), FeAS-induced phosphorylation of p38MAPK was considerably suppressed and the annexin V-positive apoptotic cell fraction was reduced from 27.6% to 9.44% (Figure 2C). Similarly, SB203580, a specific inhibitor of p38-MAPK, reduced the apoptotic fraction to 13.2% in cells exposed to FeAS (Figure 2C). Consequently, the growth inhibition observed in FeAS-treated cells was prevented by treatment with either NAC or SB203580 (Figure 2D). These results indicate that ROS are involved in FeAS-induced p38MAPK activation and that p38MAPK is a major executer of FeAS-induced growth inhibition and cell death.

### 3.2. FeAS Reduces LSK Cells Formation and Inhibits the Growth of Murine BM Cells

**Figure 3:**
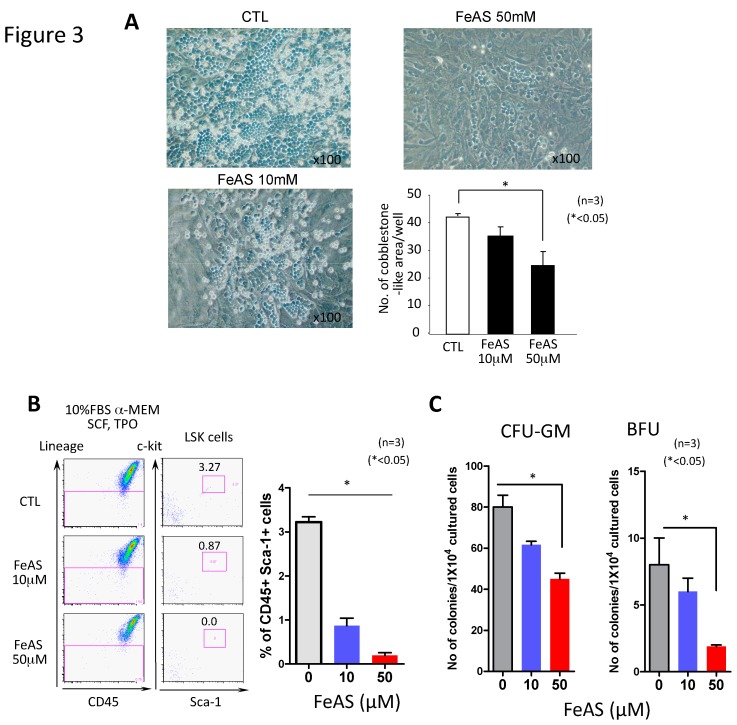
FeAS inhibits hematopoietic cell maintenance in co-culture system with MS5 stroma cells. (**A**) LSK cells (100 cells/well) were plated on pre-established monolayers of MS5 cells in a 24-well plate. After 3 or 4 days of culture in 10% FBS αMEM containing SCF and TPO (100 ng/mL each) with or without FeAS, the numbers of cobblestone-like areas were counted. Pictures of representative cobblestone-like areas from three independent experiments are shown. Original magnification: 100× (**B**) LSK cells were cultured on MS5 cells for 7 days with or without FeAS and subjected to flow cytometry. Cells were stained with anti-Lin, anti-Sca-1, and c-Kit antibodies and analyzed using a FACS Canto II. Numbers in the boxes are the percentages of each fraction. Representative flow cytometry data from one experiment are shown (left panel) and summarized data (mean ± SD) from three independent experiments is shown in the right panel (* *p* < 0.05). (**C**) Primary BM MNCs were cultured on MS5 with or without FeAS for 7 days and colony assays were performed. All cultures were conducted in triplicate and BFU and CFU-GM colonies were visualized, identified and scored in a light microscope after 10 days of culture. Results are shown as mean ± SD (*n* = 3, * *p* < 0.05).

To examine the effects of FeAS on hematopoiesis under more physiological conditions, we co-cultured murine BM MNCs or LSK cells with the MS5 stromal cell line, which is known to support the growth and survival of hematopoietic stem/progenitor cells through cytokine production and cell-to-cell interactions. After 7 days of co-culture, immature hematopoietic cells tend to grow forming cobble stone-like areas in the proximity to MS5 cells and more mature cells emerge as non-adherent cells. When FeAS was added to this co-culture system, cobble stone-like areas close to MS5 cells were substantially suppressed and the number of suspension cells decreased as well (Figure 3A). Similar results were obtained when hematopoietic stem cells (BM MNCs or LSK cells) were co-cultured with the stromal cell line OP9 cells (data not shown). Of note, as shown in Figure 3B, FeAS prevented the generation of LSKs (c-Kit+ Sca-1+) from BM MNCs (3.27% in untreated cells vs. 0% in cells treated with 50 μM of FeAS).

Next, we performed colony assays with MNCs cultured in semisolid medium with or without FeAS for 7 days, and found that the numbers of CFU-Mix, BFU-E, and CFU-GM were reduced upon FeAS treatment, implying that FeAS treatment reduces hematopoietic stem/progenitor cells (Figure 3C).

**Figure 4:**
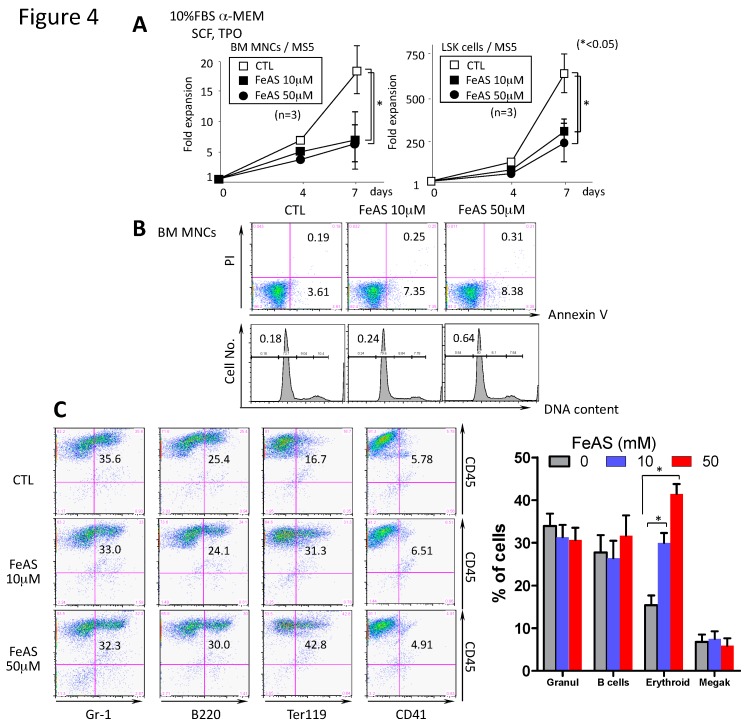
FeAS inhibits the growth of murine BM cells and modulates lineage distribution after culture with MS5 cells. (**A**) BM MNCs (1 × 10^4^ cells) or LSK cells (100 cells/well) were co-cultured with MS5 cells under the indicated conditions for 7 days. Cell proliferation was determined by counting the total viable cells using the ATP assay. The data shown are representative of three independent experiments (* *p* < 0.05). (**B**) The proportion of apoptotic cells was determined with annexinV/PI-staining. The DNA content of cells cultured for 7 days was examined by PI staining. The proportion of cells in annexin V+ and the sub-G1 fraction is indicated, respectively. (**C**) After 7 days of culture, specific antibodies were used to characterize the surface phenotype of cultured LSK cells as indicated. Representative fluorescence-activated cell sorting data from one experiment are shown (left panel) and summarized data (mean ± SD) from three independent experiments is shown in the right panel (* *p* < 0.05) (Gr-1+ granulocytes, B220 B cells, Ter119 erythroid cells and CD41 megakaryocytes).

We also analyzed the effects of FeAS on the growth of murine BM MNCs and LSK cells in this co-culture system. As observed in the liquid cultures (Figure 1A,B,C), the total number of viable cells was reduced in response to FeAS treatment, although no dose dependent inhibitory effects were observed under these culture conditions (Figure 4A). In addition, contrary to the observed in liquid culture conditions (as shown in Figure 2A), neither an increase in the proportion of Annexin V-positive nor subdiploid apoptotic cells were observed in response to FeAS treatment (Figure 4B), indicating that in the co-culture system the FeAS-induced reduction of viable cell number was mainly due to inhibition of cell proliferation rather than by inducing apoptosis or cell death.

To examine whether FeAS treatment influences terminal differentiation of hematopoietic cells, we cultured LSK cells with MS5 cells for 7 days and observed that 35.6% of the cultured cells were Gr-1^+^ granulocytes, 25.4% were B220^+^ B lymphocytes, 16.7% were Ter119^+^ erythroid cells, and 5.78% were CD41^+^ megakaryocytes. Although the proportions of in vitro generated granulocytes, B lymphocytes, and megakaryocytes did not change, regarding the presence of FeAS in the culture medium, the proportion of erythroid cells was significantly higher (*p* < 0.05) in cultures treated with FeAS, increasing from 16.7% in untreated cells to 24% and 42.8% in cells cultured in the presence of 10 and 50 μM of FeAS, respectively (Figure 4C).

### 3.3. FeAS Inhibits Terminal Differentiation of Erythroid Cells but not of Myeloid/macrophages or Megakaryocytes

**Figure 5:**
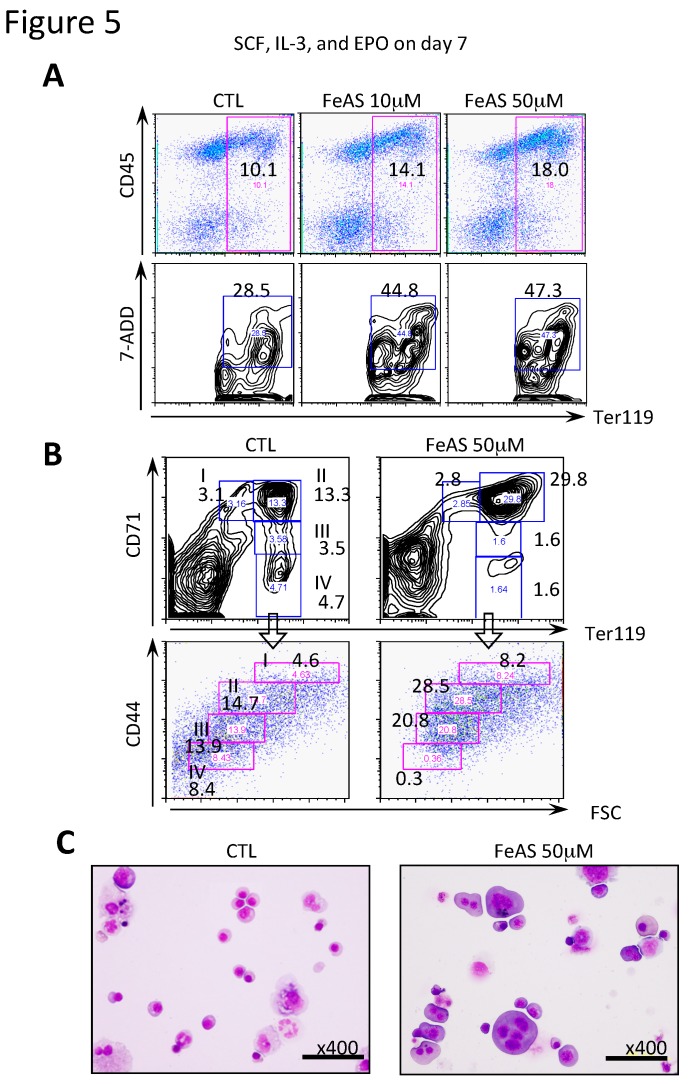
FeAS interferes with the development of erythroid cells with severe morphologic abnormalities. (**A**) Flk-1^+^ cells were induced to develop into erythroid cells using the OP9 system as described in Materials and Methods. After 7 days of culture with the indicated cytokine cocktail in the presence or absence of FeAS, the expression of CD45 and Ter119 on the cultured cells was examined by flow cytometry analysis. Apoptotic cells in the erythroid lineage were detected as Ter119^+^7-AAD^+^ cells, and the percentage of these cells is indicated. (**B**) Erythroblasts at different maturation stages were identified by double staining with FITC-conjugated anti-TER119 and APC-conjugated anti-CD44 Abs. Plots of CD44 vs. forward scatter (FSC) for TRE119-positive cells are shown. After cells were cultured as described above, the expression of CD71 and Ter119 was examined by flow cytometry analysis. The percentages of cells in fractions I to IV are indicated. I (Ter119^low^CD71^+^) corresponds to ~CFU-E; II (Ter119^+^CD71^+^) to proerythroblasts; III (Ter119^+^CD71 low) to erythroblasts; IV (Ter119^+^CD71^−^) to erythrocytes. (**C**) After 11.5 days of culture in the absence or presence of 50 μM FeAS, cultured cells were subjected to morphological analysis.

To analyze the effects of iron overload on the development of hematopoietic cells, we employed the OP9 system. In this system, after coculture with OP9 cells in the absence of LIF, ES cells differentiate into Flk-1^+^ hemangioblasts on day 4.5, which then develop into hematopoietic cells or endothelial cells. Flk-1^+^ cells then differentiate into definitive hematopoietic stem/progenitor cells on day 8.5 [31]. When 10 μM FeAS was added to the culture medium on day 4.5, the development of CD117 (c-Kit)^+^ hematopoietic progenitor cells was severely impaired (data not shown). Thus, we added FeAS on day 6.5 and examined its effects on the terminal differentiation of hematopoietic cells. When FLK-1^+^ control cells were cultured with SCF, IL-3, and EPO for 7 days, 10.1% of the controls became Ter119^+^ erythroid cells and this population increased to 14.1% in response to 10 μM FeAS and 18.0% with 50 μM FeAS (Figure 5A). However, the 7-ADD^+^ apoptotic fraction simultaneously increased from 28.5% to 47.3% in Ter119^+^ erythroid cells treated with 50 μM FeAS (Figure 5A).

To quantitatively assess the maturational stages of differentiating erythroblasts, we evaluated the expression of Ter119 and CD71 (transferrin receptor 1) and CD44 using flow cytometry in cultured cells, since these surface markers have been shown to correlate with different maturation stages of murine erythroblasts [32]. In untreated cultures, erythroid differentiation proceeded clockwise from population I to IV and normal orthoerythroblasts and erythrocytes developed from Flk-1^+^ cells, however, significantly higher numbers of cells in region II were observed in cells exposed to FeAS (Figure 5B), thus supporting the idea that erythroid maturation from proerythroblast to immature erythroblast is impaired in FeAS treated cells.

In addition, morphologic analysis using giemsa staining revealed that most of the erythoblasts showed megaloblastic changes, and some of these had multinuclei (Figure 5C, right panel). Taken together, these findings indicate that FeAS promotes maturation of erythroid cells up to erythloblasts. However, these erythroblasts exhibit dysplastic changes, fail to complete terminal differentiation, and consequently undergo cell death.

**Figure 6:**
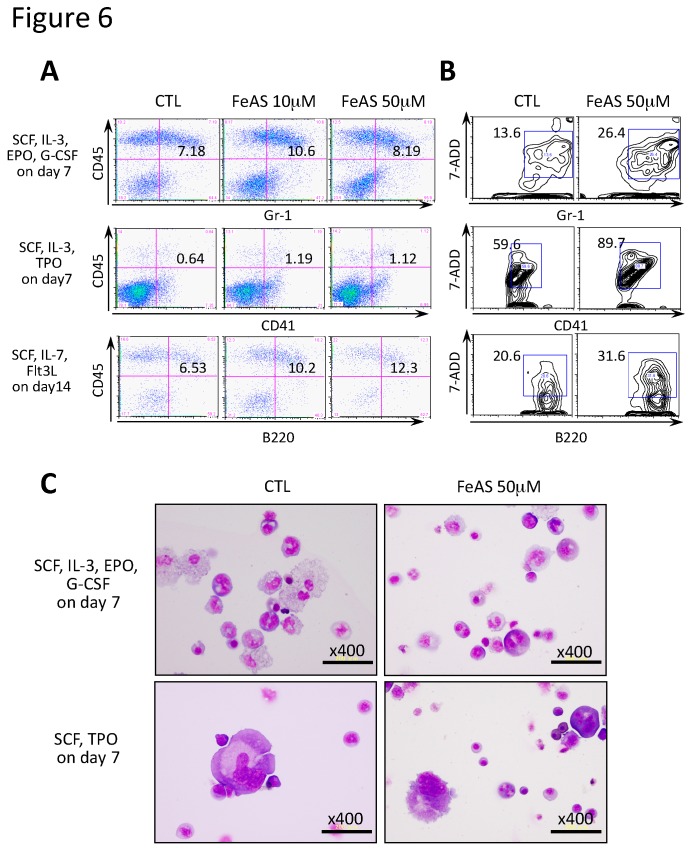
FeAS shows little influence on the development of myeloid cells, megakaryocytes, and B lymphocytes. (**A**,**B**) Myeloid cells, megakaryocytes, and B lymphocytes were induced to develop under the indicated conditions using the OP9 system, and the respective lineage markers, Gr-1, CD41 and B220, were examined by flow cytometry analysis. Reactivity to 7-AAD was also analyzed by flow cytometry. The percentage of each fraction is indicated. (**C**) The morphology of granulocytes and megakaryocytes treated with FeAS obtained by the above-described culture conditions were analyzed.

We also analyzed the effects of FeAS on the development of myeloid cells, megakaryocytes, and B lymphocytes by culturing FLK-1^+^ cells with the appropriate cytokine cocktail to induce differentiation. The proportion of Gr-1^+^ cells in FeAS-treated cultures was hardly affected when compared to controls (50 μM FeAS, 8.19% vs. CTL, 7.18%), while FeAS increased the proportion of CD41^+^ megakaryocytes from 0.64% to 1.12%, and that of B220^+^ B lymphocytes from 6.53% to 12.3% (Figure 6A). In addition, the dead cell fractions were also increased upon FeAS treatment in granulocytes (from 13.6% in CTL to 26.4% with FeAS-treated cells), as well as in megakaryocytes (from 59.6% to 89.7%), and in B lymphocytes (from 20.6% to 31.6%) (Figure 6B). On the other hand, FeAS treatment did not cause dysplastic changes nether in myeloid cells (Figure 6C, upper panel) nor in megakaryocytes (Figure 6C, lower panel).

### 3.4. Effects of FeAS on the Functions of Stromal Cells

**Figure 7:**
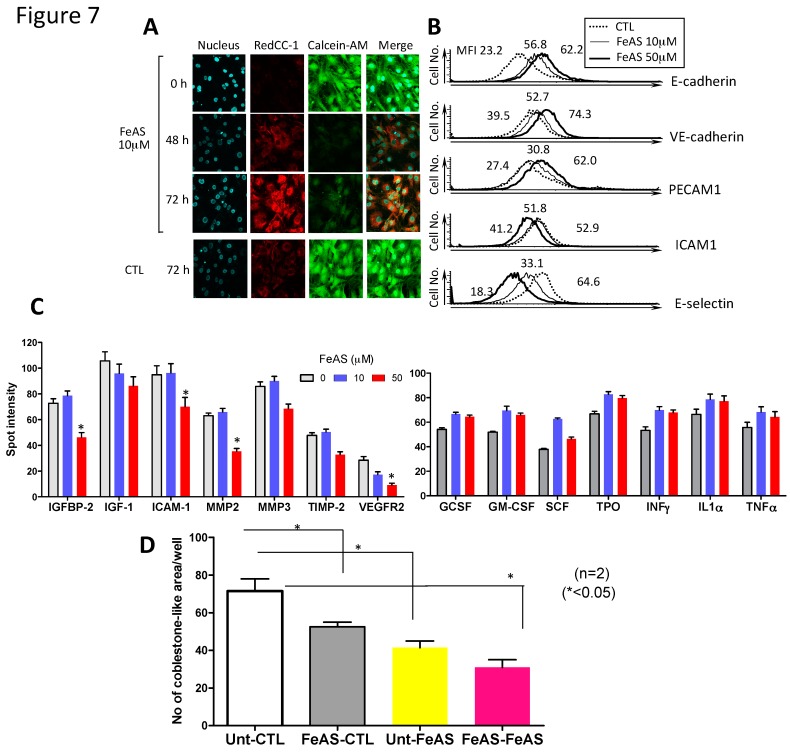
FeAS affects the expression of adhesion molecules on MS5 stroma cells and alters the profile of cytokine production. (**A**) After culture with or without FeAS, MS5 were stained as indicated and analyzed by fluorescence microscopy. Total ROS were detected with Red CC-1, intracellular labile iron with Calcein-AM, and nuclei with Hoechst 33,342. (**B**) After MS5 cells were cultured with or without FeAS for 48 h, cells were stained with the indicated Abs. Representative flow cytometry histograms from three independent experiments are shown. (**C**) Conditioned media were analyzed with a cytokine array and the accompanying kit reagents and protocol. Conditioned media from FeAS-treated (10 or 50 μM) or untreated MS5 cells (CTL) was collected after 48 h. of culture. Medium supplemented with 10% FBS αMEM was used as a negative control (NC). The assay is arranged in a 14 × 10 grid (Array 3) and a 12 × 8 grid (Array 4), with the configuration shown in Supplementary Data (Figure S1A,B). Summarized densitometry data (mean ± SD) from three independent experiments is shown in the right panel (* *p* < 0.05). (**D**) MS5 cells were cultured in the presence or absence of FeAS for 48 h, and co-cultured with LSKs with or without FeAS for another 48 h and the number of cobblestone-like areas seen in each well were counted under light microscopy. Summarized data (mean ± SD) from two independent experiments is shown (* *p* < 0.05). Unt-FeAS: FeAS was present in the culture medium only when MS5 cells were co-cultured with LSK; FeAS-CTL: FeAS was present in the culture medium when MS5 cells were cultured alone but not when they were co-cultured with LSK; FeAS-FeAS: FeAS was present in the culture medium in both, when MS5 cells were cultured alone and when they were co-cultured with LSK cells; and Unt-CTL: FeAS was not present in the culture medium neither when MS5 cells were cultured alone nor when they were co-cultured with LSK.

Next, we examined the effect of FeAS on stromal cells, since they are important component of bone marrow niche and is a crucial regulator of hematopoiesis [33]. When MS5 cells were cultured in the absence of FeAS, the fluorescence intensity of Calcein-AM staining was maintained at strong levels even after 72 h of culture, and ROS were scarcely detected (Figure 7A). In contrast, when FeAS was added to the culture medium, the fluorescence intensity of Calcein-AM severely decreased after 48 to 72 h, and ROS gradually increased, starting at 48 h and onwards. These results indicate that FeAS was incorporated into MS5 cells and produced ROS.

As BM stromal cells are known to support the growth and survival of hematopoietic stem/progenitor cells through cell-to-cell interactions [33], we examined the effects of FeAS on the expression of adhesion molecules on MS5 cells. As shown in Figure 7B, FeAS treatment augmented the expression of E-cadherin, VE-cadherin, and PECAM1, and suppressed that of ICAM1 and E-selectin. This result indicates that the interaction between hematopoietic stem/progenitor cells and BM stromal cells may be modified, to some extent, by iron overload. We further examined whether FeAS treatment affects cytokine production of MS5 cells. For this purpose, we cultured MS5 cells with or without FeAS for 48 h, collected the supernatant, and analyzed the media using a cytokine antibody array (Figure 7C). Compared to the control medium containing 10% FBS (NC), untreated control MS5 cells were found to secrete CXCL16, IL-1α, LIX, MCP-1, MIP-1, SDF-1α, and TIMP-1 (using Array 3), and IGFBP-2, IGF-1, and Osteopontin (using Array 4) (Supplementary Figure 1A,B). When FeAS was added at 10 or 50 μM, fewer amounts of insulin-Like Growth Factor-Binding Protein 2 (GFBP-2), insulin-Like Growth Factor 1 (IGF-1), Matrix metalloproteinase 2 and 3 (MMP2, MMP3), tissue inhibitor of metalloproteinase 2 (TIMP-2), soluble intracellular adhesion molecule 1 (ICAM1), and vascular endothelial factor receptor 2 (VEGFR2) were secreted. In contrast, we detected an apparent increase of TPO, SCF, G-CSF, GM-CSF, IL-1α, interferon γ (IFN-γ), and TNF-α in response to FeAS treatment. Finally, to evaluate the significance of these changes in stromal cell function, we cultured MS5 in the presence or absence of FeAS for two days and then they were co-cultured with LSKs with or without FeAS and the number of cobblestone-like areas were determined.

As shown in Figure 7D, the formation of cobblestone-like areas was suppressed when stromal cells (MS5 cells) were pretreated with FeAS. As IGF-1, SDF-1β, TPO, SCF, G-CSF, GM-CSF, IL-1β, IFN-γ, and TNF-α are involved in the regulation of growth differentiation and survival of hematopoietic cells from immature to differentiated cells [34], we believe that excessive iron may influence hematopoiesis by modifying the function of BM stromal cells, especially in the regulation of the fate of HSCs (i.e., self-renewal vs. differentiation). Notably, the inhibitory effects of iron overload were more severe when both stromal cells only, or stromal cells + LSKs, were exposed to FeAS (Figure 7D), indicating that iron overload may interfere with hematopoiesis by affecting both HSC and stromal cells.

## 4. Discussion

Iron overload is a common feature of several blood disorders, including diseases associated with primary iron overload (hereditary hemochromatosis) and various disorders that require chronic blood transfusion, such as aplastic anemia, thalassemia and sickle cell disease.

In this study, we found that excessive iron induces deleterious effects in hematopoietic stem/progenitor cells as well as in differentiated hematopoietic cells, likely through ROS accumulation. Notably, the results shown here are not consistent with the observation that patients with hereditary hemochromatosis do not exhibit major abnormalities in hematopoiesis despite the excessive iron accumulation during the course of the disease [35]. One possible reason for that discrepancy is that, in vivo, hematopoietic stem cells are more effectively protected by the bone marrow microenvironment, which is not the case in the in vitro system (co-culture with stromal cell lines) utilized in this study. Alternatively, the accumulation of NTBI and LIP may not be so severe in patients with hemochromatosis since the gradual progression of iron overload during the course of the disease may allow the development of adaptive or protective mechanisms.

Chai and colleagues previously reported the deleterious effects of iron overload on bone marrow hematopoiesis. In their study, mice exposed to high levels of iron-dextrin resulted in impaired hematopoiesis and those effects were in part, mediated via elevated ROS production, although the precise mechanisms of those inhibitory effects remained unresolved [36]. In the present study, using various in vitro culture systems, we have confirmed that hematopoiesis is severely impaired when hematopoietic stem cells are exposed to high amounts of iron and we have demonstrated the essential role of ROS induction and the MAPK38 hyperactivation in the inhibition of hematopoiesis resulting from the exposure of hematopoietic stem cells to high concentrations of iron.

In this study, we found that FeAS inhibited the maturation of erythroid cells at the stage of proerythroblasts to immature erythroblast and induced cell death in these cells. This result is largely consistent with the phenotype of the heme transporter FLVCR knockout mouse that has been reported before [37], where conditional FLVCR knockout mice exhibit severe anemia due to the maturation arrest of erythroid cells at the CD71^+^Ter119^+^ stage. In our study, the remarkable sensitivity of erythroid cells to the deleterious effects of iron overload contrasts with the apparent resistance of others cells lineages including myeloid cells, megakaryocytes, and B lymphocytes which were scarcely affected by FeAS treatment, a finding that is consistent with the phenotype of FLVCR knockout mice, which did not show an apparent abnormality in these cell lineages [37]. The results are also consistent with a recent study reporting that iron accumulation in hematopoietic stem cells from FBXL5 deficient mice, resulted in impaired hematopoiesis bud did not substantially affect more differentiated hematopoietic cells [22] and are also in agreement with a recent study reporting that iron overload, induced via iron dextran administration, impaired erythroid cells development in a mouse model of myelodysplastic syndrome [38]. Together, these results indicate that although an appropriate amount of iron is required for erythroid cells to execute Hb synthesis, excessive iron impairs full maturation and induces apoptosis in these cells and indicate that the susceptibility to apoptosis caused by excessive iron is likely cell lineage-dependent.

In this study, we assessed the direct effects of labile iron (Fe^2+^) in the form of FeAS on in vitro hematopoiesis studies in co-culture with embryonic stem cell lines OP9 and E14tg2a cells and utilized a low concentration of ascorbic acid to maintain iron in its ferrous state. FeAS was used at similar concentrations utilized in previous studies [39], with some researchers reporting no major cell toxicity in other cellular systems. For example, Hsieh et al. observed that ferrous iron-induced apoptosis in cultured human cardiomyocytes only when it was utilized at very high concentrations (0–1000 μg/mL) [40]. On the other hand, the sustained exposure of the myelomonocytic cell line THP-1 to high concentrations of Fe^2+^ (100 µM) did not impair viability and did not change cellular morphology [41]. The fact that in our study only the erythroid lineage was affected by FeAS, indicates that the dose utilized is not toxic for all cells. In addition, although cells exposed to high concentrations of ascorbic acid (>5 mM) can undergo apoptosis [42], ascorbic acid concentrations utilized in our experimental conditions are not toxic and did not affect the cytokine-dependent growth of HSCs.

Accumulating evidence suggests that the bone marrow microenvironment, or the stem cell niche, plays an important role in maintaining hematopoietic stem/progenitor cells through the secretion of various cytokines and by direct cell-to-cell interactions [33,43]. In this study we found that, in addition to the direct toxic effects on hematopoietic cells, excessive iron may simultaneously alter the function of BM stromal cells, thereby injuring the total hematopoietic system. In fact, here we found that the expression levels of several cell adhesion molecules in MS5 cells was significantly altered by FeAS treatment (upregulation of E-cadherin, VE-cadherin, and PECAM1; downregulation of ICAM1 and E-selection). Furthermore, in screening cytokine production of MS5 cells, we found that although FeAS did not induce an apparent change in the expression of several cytokines and chemokines, such as CXCL16, IL-1β, LIX, MCP-1, MIP-1α, SDF-1α, TIMP-1 and Osteopontin, it reduced the secretion of IGFBP-2, IGF-1, MMP2, MMP3 and the soluble form of ICAM1. In contrast, it augmented the secretion of IFN-γ, TNF-α, TPO, SCF, G-CSF, GM-CSF and IL-1α. Since a regulated balance between self-renewal and differentiation is required for the maintenance of hematopoietic stem cells, the FeAS-induced changes in the cytokine profiles likely have dramatic effects on hematopoiesis [34]. This is important since various cytokines can regulate hematopoiesis and especially IFN-γ and TNF-α are well-known to inhibit directly inhibit hematopoietic stem cells [44,45]. Furthermore, since immature progenitor cells and more differentiated cells are also targets of these cytokines [45,46], these alterations would also affect terminal differentiation of hematopoietic cells from hematopoietic stem/progenitor cells.

As an essential component of the bone marrow microenvironment, bone marrow stromal cells play critical roles in hematopoiesis by regulating the stem cell niche, secreting cytokines and growth factors that promotes the differentiation and maturation of different cell lineages. Altered stromal cells functions have been documented in various blood disorders, including leukemia, aplastic anemia MDS. For example, excess production of inhibitory cytokines contributes to ineffective hematopoiesis in MDS [47] and several studies have shown that myelosuppressive and proinflammatory cytokines such as transforming growth factor-β (TGF-β), IL-6 and IFN-γ and IFN-α are elevated in the serum of MDS patients [48,49]. As the secretion of TNF-α and IFN- α from MS5 cells was upregulated by FeAS in this study, we speculated that iron overload may modify the function of stromal cells not only in the normal state but also in the pathologic state, thereby affecting the pathophysiology and severity of the background disease.

IGFBP2 and IFG-1, which were downregulated in the presence of FeAS in our experiments, have been found to stimulate the survival, proliferation and cycling of HSCs [50] and specially IGF1 promotes osteoblastic niche expansion and HSC engraftment in the bone marrow [51]. In addition, bone marrow stromal cells that are deficient for IGFBP2 have significantly decreased ability to support the expansion of repopulating HSCs [52].

Bone marrow mesenchymal stem cells (BMMSC), an important component of the bone marrow microenvironment and regulator of hematopoiesis, undergo programmed cell death and necrosis when exposed to excessive iron concentrations [53] and in mice exposed to high doses of iron dextran, impaired osteogenic differentiation of BMMSC along with reduced levels of VEGF and CXCL12 were observed [54]. On the other hand, osteoblasts, which play crucial roles in the maintenance of HSC pool, bone marrow microenvironment and ultimately regulate hematopoiesis [55], are also harmed by iron overload, as shown in a recent study reporting that iron overload impairs the growth and function of osteoblasts via inhibition of PI3K/AKT signal [39]. These observations add a new layer of complexity to the mechanisms by which excessive iron can impair hematopoiesis.

## 5. Conclusions

In summary, here we showed that excessive iron impairs hematopoiesis through direct effects on hematopoietic cells and indirect effects mediated by stromal cells. Further studies to analyze the effect of iron overload on normal and mutated hematopoietic cells would clarify the roles of iron chelation therapy for patients with iron overload.

## Supplementary Materials

The following are available online at https://www.mdpi.com/2073-4409/8/3/226/s1. Figure S1: The profile of cytokine production in the media culturing MS5 cells.
