# Supplementary material for: Excessive Reactive Iron Impairs Hematopoiesis by Affecting Both Immature Hematopoietic Cells and Stromal Cells
Source: Cells. 2019 Mar 8;8(3):226. doi: 10.3390/cells8030226 (PMC6468739; doi:10.3390/cells8030226)
Supplement: Supplementary file 1 [file cells-08-00226-s001.pdf]

Figure S1

A

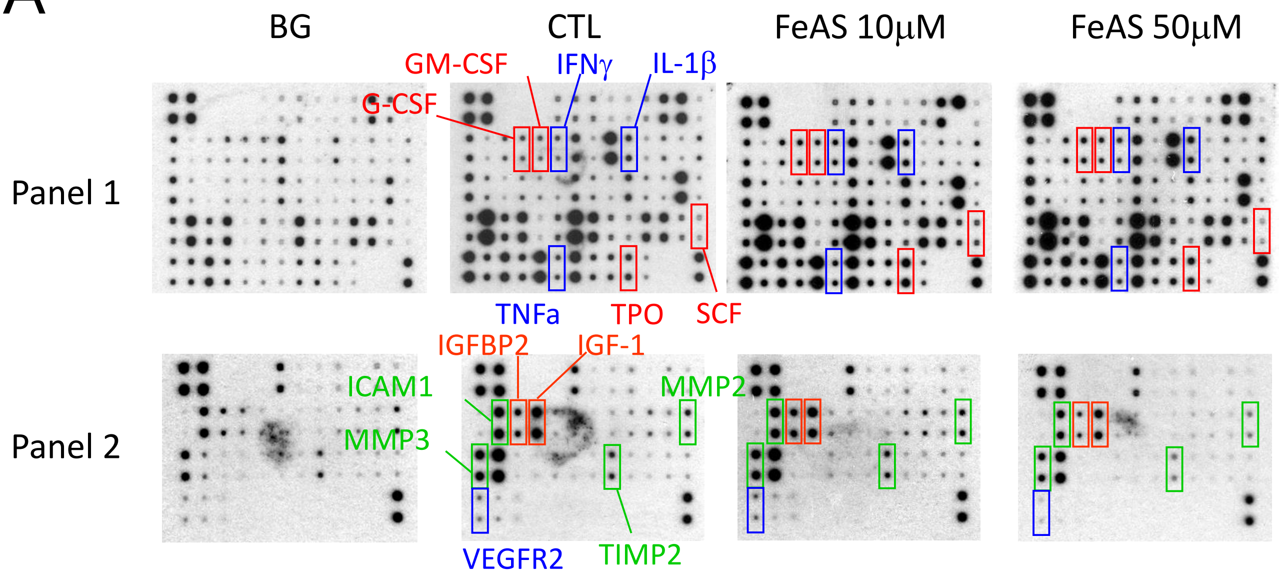

B

RayBio® Mouse Cytokine Antibody Array C Series 1000

Combine Mouse Cytokine Antibody Array 3 and 4 to detect 96 cytokine experssion in one experimant

RayBio® Mouse Cytokine Antibody Array 3

Detect **62** cytokines and other proteins in one experiment

	A	B	C	D	E	F	G	H	I	J	K	L	M	N
1	POS	POS	NEG	NEG	Blank	Axl	BLC	CD30 L	CD30 T	CD40	CRG-2	CTACK	CXCL16	Eotaxin
2	POS	POS	NEG	NEG	Blank	Axl	BLC	CD30 L	CD30 T	CD40	CRG-2	CTACK	CXCL16	Eotaxin
3	Eotaxin-2	Fas Ligand	Fractalkine	GCSF	GM-CSF	IFN γ	IGFBP-3	IGFBP-5	IGFBP-6	IL-1α	IL-1 beta	IL-2	IL-3	IL-3 Rb
4	Eotaxin-2	Fas Ligand	Fractalkine	GCSF	GM-CSF	IFN γ	IGFBP-3	IGFBP-5	IGFBP-6	IL-1α	IL-1 beta	IL-2	IL-3	IL-3 Rb
5	IL-4	IL-5	IL-6	IL-9	IL-10	IL-12 p40/p70	IL-12 p70	IL-13	IL-17	KC	Leptin R	Leptin	LIX	L-Selectin
6	IL-4	IL-5	IL-6	IL-9	IL-10	IL-12 p40/p70	IL-12 p70	IL-13	IL-17	KC	Leptin R	Leptin	LIX	L-Selectin
7	Lymphotactin	MCP1	MCP-5	M-CSF	MIG	MIP-1α	MIP-1γ	MIP-2	MIP-3 β	MIP-3α	PF-4	P-Selectin	RANTES	SCF
8	Lymphotactin	MCP1	MCP-5	M-CSF	MIG	MIP-1α	MIP-1γ	MIP-2	MIP-3 β	MIP-3α	PF-4	P-Selectin	RANTES	SCF
9	SDF-1α	TARC	TCA-3	TECK	TIMP-1	TNFα	sTNF RI	sTNF RII	TPO	VCAM-1	VEGF	Blank	Blank	POS
10	SDF-1α	TARC	TCA-3	TECK	TIMP-1	TNFα	sTNF RI	sTNF RII	TPO	VCAM-1	VEGF	Blank	Blank	POS

RayBio® Mouse Cytokine Antibody Array 4

Detect 34 cytokines and other proteins in one experiment

	A	B	C	D	E	F	G	H	I	J	K	L
1	POS	POS	NEG	NEG	BLANK	bFGF	DPPIV/CD26	Dtk	E-Selectin	Fcγ RIIB	Flt-3 Ligand	GITR
2	POS	POS	NEG	NEG	BLANK	bFGF	DPPIV/CD26	Dtk	E-Selectin	Fcγ RIIB	Flt-3 Ligand	GITR
3	HGF R	ICAM-1	IGFBP-2	IGF-I	IGF-II	IL-15	IL-17B R	IL-7	I-TAC	Lungkine	MDC	MMP-2
4	HGF R	ICAM-1	IGFBP-2	IGF-I	IGF-II	IL-15	IL-17B R	IL-7	I-TAC	Lungkine	MDC	MMP-2
5	MMP-3	Osteopontin	Osteoporotegerin	Pro-MMP-9	Resistin	Shh-N	Thymus CK-1	TIMP-2	TRANCE	TROY	TSLP	VEGF R1
6	MMP-3	Osteopontin	Osteoporotegerin	Pro-MMP-9	Resistin	Shh-N	Thymus CK-1	TIMP-2	TRANCE	TROY	TSLP	VEGF R1
7	VEGF R2	VEGF R3	VEGF-D	BLANK	BLANK	BLANK	BLANK	BLANK	BLANK	BLANK	BLANK	POS
8	VEGF R2	VEGF R3	VEGF-D	BLANK	BLANK	BLANK	BLANK	BLANK	BLANK	BLANK	BLANK	POS

* For use with serum, plasma, condition medium, urine and all other body fluids, cell lysates and certain tissue lysates samples
